# Study participants incentives, compensation and reimbursement in resource-constrained settings

**DOI:** 10.1186/1472-6939-14-S1-S4

**Published:** 2013-12-19

**Authors:** Takafira Mduluza, Nicholas Midzi, Donold Duruza, Paul Ndebele

**Affiliations:** 1 College of Health Sciences, University of KwaZulu-Natal, Durban, South Africa; 2 University of Zimbabwe, P.O. Box MP 167, Mount Pleasant, Harare, Zimbabwe; 3 Research Council of Zimbabwe, P.O. Box CY 294, Causeway, Harare, Zimbabwe; 4 Zimbabwe Revenue Authority, PO Box CY 803, Causeway, Harare, Zimbabwe; 5 Medical Research Council of Zimbabwe, P.O. Box CY 573, Causeway, Harare, Zimbabwe

**Keywords:** study participants, incentives, compensation, reimbursement, resource-constrained

## Abstract

**Introduction:**

Controversies still exists within the research fraternity on the form and level of incentives, compensation and reimbursement to study participants in resource-constrained settings. While most research activities contribute significantly to advancement of mankind, little has been considered in rewarding directly the research participants from resource-constrained areas.

**Methods:**

A study was conducted in Zimbabwe to investigate views and expectations of various stakeholders on study participation incentives, compensation and reimbursement issues. Data was collected using various methods including a survey of about 1,008 parents/guardians of school children participating in various immunological cohort studies and parasitology surveys. Community advisory boards (CABs) at 9 of the sites were also consulted. Further, information was gathered during discussions held at a basic research ethics training workshop. The workshop had 45 participants that including 40 seasoned Zimbabwean researchers and 5 international research collaborators.

**Results:**

About 90% (907) of the study participants and guardians expected compensation of reasonable value, in view of the researchers' value and comparison to other sites regardless of economic status of the community. During discussion with researchers at a basic ethics training workshop, about 80% (32) believed that decisions on level of compensation should be determined by the local research ethics committees. While, the few international research collaborators were of the opinion that compensation should be in accordance with local guidelines, and incentives should be in line with funding. Both the CAB members and study participants expressed that there should be a clear distinction between study incentive and compensation accorded to individual and community expectations on benefits from studies. However, CABs expressed that their suggestions on incentives and compensation are often moderated by the regulatory authorities who cite fear of unknown concerns.

**Conclusion:**

Overall, both personal and community benefits need to be considered colectively in future studies to be conducted in resource-constrained communities. There is projected fear that recruitment in future may be a challenge, now that almost every community, has somehow been reached and participated in some form of studies. A major concern on reimbursement, compensation or incentives should be internationally pegged regardless of different economic status of the individuals or communities where the study is to be conducted.

## Background

Biomedical studies are well known to add scientific solutions to problems bedeviling mankind, animals and their environment [1-3]. Through conducting research activities, some benefits are also realized by individuals and communities worldwide. Research programs assist in finding out new ways for treatment, to solve some medical problems and to improve the health standards of living for humans [4]. However, with the benefits realized at multiple-levels, concern is raised where the subjects of the research activities, who are at the core of the programme, rarely obtain any visible individual benefits [5].

Research participants indulge in different study protocols for different reasons. Sometimes the main driving force is beyond the participants' control [6]. While currently, the international research foundations and organizations are struggling to rationalize participation, in an effort to tame the research jungle [7,8]. However, at individual levels there are a couple of questions that go unanswered for the participants who are right at the bottom of the planning and the research protocol hierarchy [5,6]. Some of the pertinent questions by the research participants that go unanswered include the following: i). What is my immediate benefit? ii). Who will benefit from this work being conducted? ii). Why are these people (researchers) using all the resources in my community and yet we have so many other problems? iii). Why my community and not that other community? iv). The researchers are here for only 2 years, then what next? v). These people are well off, better than anyone in our community, so they must be benefiting from the activities?

While researchers are well aware of the main goal(s) including on how to achieve these through data/sample gathering, a lot need to be understood on the study participants and their feelings. Further, it is reasonable not to assume but to become part of the community and feel from within what the participants expect from taking part in studies and providing their biological specimens. Some progress has been achieved along these lines with moderate consideration on certain aspects that affect study participants in the form of repayment or reimbursement, which is replacement of what could have been lost or what could have been gained during the time the participant is involved in the research activities [9-13]. While there are other forms that have been coined into studies that include giving out incentives; that represents something that motivates or encourages participation. Incentives are rarely permitted by study regulators for reason still unclear to many researchers, especially when an incentive is considered as payment or a concession to stimulate greater participation, this is regularly not permitted by most in-country study regulatory agents and in certain instances by research groups [5]. Where the commonly applied consideration of benefits to study participation by researchers is compensation, representing something that makes up for an undesirable or unwelcome state of affair or this can be something, typically money awarded to someone as a recompense for loss, injury or suffering.

Most research activities involve an intertwined relationship between different players working together to achieve a common goal. In the case of simple investigative research emanating from a researcher, there are several regulatory authorities that may be responsible for giving approval for sample shipping or exchange, drug use and other commonly regulated activities [14]. Additionally, there could be some local authorities that are responsible for over sight within certain areas and research institutions. Key to the regulatory and monitoring biomedical research involving humans is the ministry of health and other ministries that deal with the public. In certain locations, there is political leadership that has control over access and running any research activities in their communities [5]. The biomedical researcher/investigator has to prepare documents that go through all the required local regulatory institutions and offices that include the ethics review board. The ethics review committees are believed to be representing the communities where research is to be conducted. The members are believed or expected to have the community at heart and to also have in-depth understanding of both traditional and cultural beliefs. In this hierarchy, the research community/participants are located right at the bottom or the receiving end. The concerns of the community or participants are assumed as represented by the structures within the areas and the regulatory authorities [5,14].

Controverses exists on the forms and levels of incentive, compensation and to lesser extent re-imbursement to study participants in resource-constrained settings. Inequity exists on addressing study participants' involvement in research studies according to economic status of the areas where the studies are conducted. Regulatory authorities need to reach a compromise, rather than dictate the level, type or amount. Currently, there is a huge demand for biomedical research or trial populations, especially in areas still developing and carrying the burden of diseases. Africa has abundant virgin testing grounds for new tools produced by biomedical and genomic revolution, and vaccines for several infections challenges. This is compelled by the great diseases burden with easy to reach sample size. Rarely are requests for conducting studies in the African populations denied due to the scarce and poor health facilities, hence communities and the leaders would be expecting access to some improved health tools and products through hosting of studies. In such areas where capacity is lacking, the biomedical research activities being conducted may seldom be observed and monitored closely by the regulatory authorities and even by CABs who may not understand the scientific implications of such studies. The participant is found to be lowly considered and rarely consulted during the design of the study protocols rather everything is assumed from consultations with ministries and regulatory authorities. Major concern is that the community settlements are often dispersed and individuals find it difficulty to have a common stand. While in recent years it has come to light that some sponsors/funders are ready to accommodate as long as proposed in the line of expenditure by the investigating team towards study participant incentives, reimbursement and even compensation, since the sponsors sometimes have commitment to the community by providing services including alleviating poverty. The whole stages of considering compensation or even rewarding study participants has never been appropriately debated taking into account the concerns of the communities. While application of ethical principles has no mathematical formula, this has to take into account various prevailing factors that include economic, social, cultural religious, civil protection systems and other relevant factors. These factors may lead to procedural differences, but the spirit of the principle remains the same.

Some unscrupulous international and local researchers take advantage of the poor and uncoordinated research systems in resource-constrained areas. Rarely would local authorities keep keen track of the activities, and such a system is bound for abuse. Further, due to rampant poverty coupled with ignorance (in a sense), and sometimes there is prevailing abundant human rights abuses; this entail disregard of community rights and respect [15-18]. Most research activities require monitoring and this decision must be reached at planning level in comparison to studies conducted in other sites in developing countries. The African health challenges expose research participants, and also including researchers and institutions to exploitation, coercion, enticement and inducement [5,19-22]. Resource constrained communities are generally deprived of most common attributes of a well-sustained and democratic societies [23,24]. The basic human rights are not observed and the individuals, probably through ignorance or due to none existence, do not have any recourse to law [23]. This aspect is not available in African communities and sometimes the poor participant living in resource-constrained community can get assistance from NGO who attempt to lobby on their behalf [25,26]. However, critically analyzing the situation reveals a couple of stages where such mishap may be avoided during the planning stages of the proposal. The sponsor/funder, investigator, regulatory authorities assume not aware of this infringement on the participants. Through activities in immunological studies and parasitology surveys conducted in Zimbabwean communities; a study was conducted to investigate views and expectations of various stakeholders on study participation incentives, compensation and reimbursement issues.

## Methods

### Study sites

Eleven communities from districts in Zimbabwe participated in the study as follows: Burma Valley, Charehwa in Mutoko, Magaya and Chigono in Murehwa, Goromonzi, Kariba, Magunje and Karoi in Hurungwe, Shamva and Trelawney [27-43]. These rural districts are situated in areas where communities survive on subsistence farming, growing maize, groundnuts, sunflowers and soya beans. While a few of the communities survive on market gardening and small-scale irrigation activities. Data from 1,008 adult participants enrolled in the 11 intervention communities of biomedical studies were included in this analysis. The observations in school children involved about 1,450 school children (95%CI: 120-173 per school) in 10 rural schools. We excluded individuals who were not willing to respond to the questionnaire at the baseline data collection points. Responding to the questionnaire at baseline indicated willingness to participate even though such individuals may not have participated at other subsequent follow-up time points.

### Data collection

Data was collected in various methods including a survey. The survey of parents/guardians of school children participating in various immunological cohort and parasitlogy survey studies. Community advisory boards (CABs) members at 9 sites in the different study communities were also consulted and lastly 45 participants that including 40 Zimbabwean researchers and 5 international research collaborators. Community based field health workers who were part of the community and were also involved in study promotion and implementation activities collected data regarding participation. The field staffs were trained in interviewing and observation techniques, data recording, and participatory community motivation approaches. The field staff recorded willing participation indicators during the days of the follow-up with a structured, observational questionnaire. In addition, field staff recorded self-reported attendance at the end of the study follow-up point through a questionnaire. These were compared to the data recorded by the research teams at the field site and in the laboratory shown by the actual presence of the collected biological sample. In order to arrive at an outcome that describes willingness to participation, we selected six complementary survey indicators that measure multiple dimensions of potential willingness (Table 1). Based on this group separation, we used characteristics of participation in the groups to describe them in meaningful, qualitative terms: Group 1 = 'willing participants', Group 2 = 'moderate participants', Group 3 = 'availability of samples as willing to participate' Group 4 encouraged participants, Group 5 = 'organized participants' and Group 6 'participation due to self/community benefits.'

**Table 1:** Indicators for study participation as classified according to groups and the rational or interpretation for analyses.

Group	Indicator	Rational and Interpretation
	Study participation indicators	
		
1	"Willing participation" Proportion of attendance during which samples were collected (as observed by community-based health staff).	Indicator for the intention to willingly participate. Indirect indicator to measure contentment and awareness.
2	"Obtaining all required samples" Proportion of samples obtained during the study (as observed by research staff).	Individuals readily available and provide the required samples. Considered to be a more reliable indicator for actual attendance than "willing participation."
3	"Samples available on incentive days" Proportion of samples obtained during which an incentive was given out (judgment by research staff after observing trends of the samples given for each study visit and at all follow-up visits).	Considered the most reliable indicator for need to be compensated. Research staff working in the community later administered questionnaire after the observations of samples available on day incentives were given out, during the time points compensation was available and days thereafter the news has filtered out of the incentive.
4	"Community Advisory Board members persuasion" the occurrence of samples during and after CABs talking to adult participants.	Indicates behavioral change due to CABs talk/chat. Reflects an increase or a decrease in number of samples during the period.
5	School children participation "Provision of blood samples" Present at school and availability of blood sample on the day bleeding is conducted.	Identifies school children providing any other none invasive samples during the study follow-up period.
6	"Treatment days" Total number of participants treated during the day's treatment was recorded.	Identifies proportion of participants with observed participation during treatment days.

### Statistical analysis

To identify patterns of study participation, we explored the quantitative distribution of study participation in terms of the six quantitative indicators (Table 1). Six differentiated groups were identified. To confirm the patterns of study participation we further examined the distribution of the willingness from samples obtained at examination day and the availability of samples, including reports from encouragement by the CABs as indicators. Willingness to participate measures in diverse communities and individual level characteristics were deduced between groups with data compared. The identified participation groups were then used in the comparison analyses. Data were analyzed using Statistical Package for Social Scientists (SPSS) version 8.0 for Windows, SPSS Inc. Chicago, USA.

### Ethical approval

The biomedical studies obtained ethical approval from the Medical Research Council of Zimbabwe and also for each area, local leaderships were consulted and permission granted [27-43]. The provincial medical and education directorates provided permission while at the community level; the community leaders and CABs were first consulted for the main biomedical studies and also accepted the study. Informed consent was obtained from community leaders, adult participants, parents or guardians of school children prior to implementation of the main biomedical projects.

## Results

The field-based monitoring staff assessed the biomedical research compliance during baseline examination and at each subsequent follow-up time point in different studies conducted over a period of 6 years, from January 2005 to December 2010. The median duration of studies was 2 years, range: 0.5 year - 4 years, in 11 community-based studies, giving a total of 26 time points. At the community level, on average there were 95 participants per site (95% CI: 73 - 117), with participation patterns observed and a questionnaire administered. A total of 9 community advisory boards were interviewed (median 8 members/community, 95% CI: 7-12). Five of the studies involved taking blood samples while the rest involved epidemiological parasitology surveys and treatment programmes.

**Figure 1:**
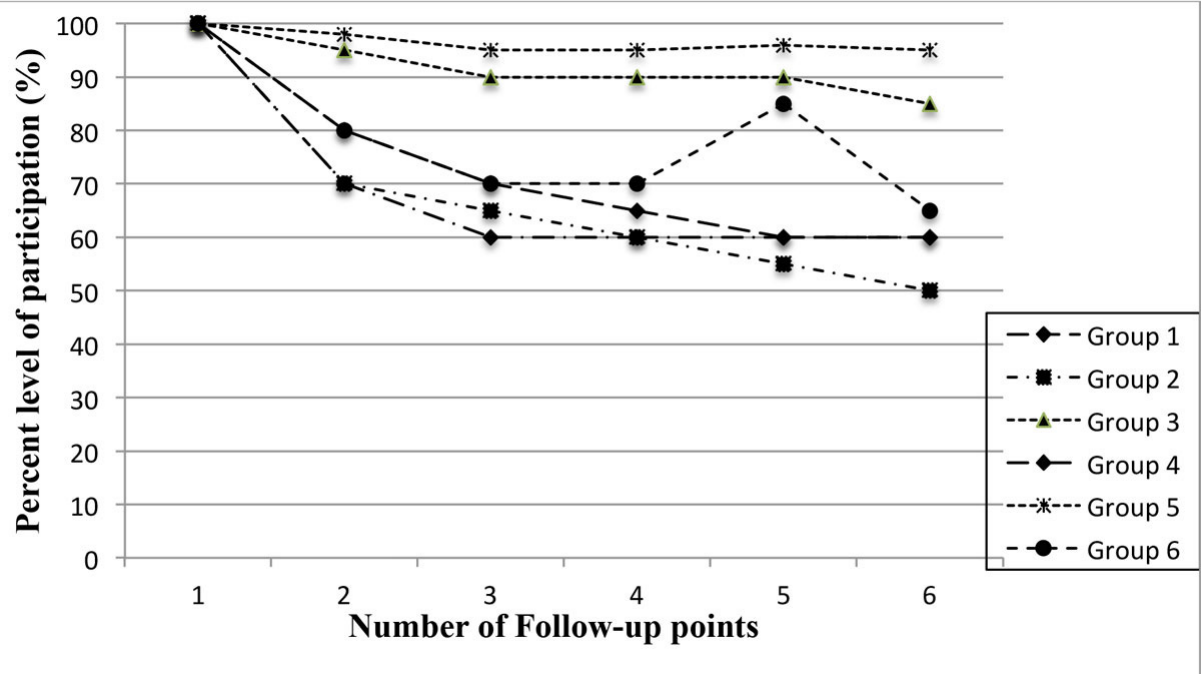
**Showing the trends in levels of participation according to groups used in the analysis at different follow-up time points for the study sites summarized**. The time points for each group may differ in year and type of study but the measurement was similar of the research subject willingness to participate.

The level of participation varied, depending on the indicator used and the source of information. The encouragement by community-based staff (CABs) led to a moderate increase of 35% from the original 25% in the different communities. Participation compliance as observed by the research staff registered an increase during the follow-up visits where treatment was provided or a token of appreciation was given, with a median proportion of 80% (IQR: 65-95) in communities assessed. After 6 years of intensive research study implementation, the questionnaire administered by the research staff assessing study participation recorded 80% of respondents reported needing personal benefit from participation, and over 80% explicitly hinting on the need for revisions in determining the benefits for study participation. During the last intensive follow-up in each community the study staff deduced a declining willingness to participate, regardless of such benefits like provision of treatment for the examined parasite (Figure 1). By the end of the study, it was revealed that compensation and incentives were constantly being indicated as the main stimulation factor for adults' participation.

Table 2 summarizes the results of the analysis, which identified six distinct participant groups based on purely on willingness to participate, and provision of all required samples as indicators. Group 5 (10 schools assessed), differed from the other groups with respect to the participants under observation as a type of a highly organized community with certain rules and regulation. While indicators 1 and 4 were more of community leaders and research staff noting upsurge of participation from records of samples obtained. These observations were noticed to decline with time into the study even though this recorded a high variability in all of the indicators as it was difficulty to base participation on the persuasion by the CABs members. Groups 2, 3 and 6 comprised indicators with the highest visible and tangible outcomes, while Group 2 with an initially high observation that declined over time, and group 4 showed a clear pattern when incentives were made available. Group 6 showed that regardless of the most important benefit to the participants of treatment available at no cost, participation was seen to decline drastically with time into the projected timeframe, an indication of probable participation fatigue. Table 2, shows the difference between groups in 6 different participation indicators (research staff-reported, community observed) and two other monitoring indicators.

**Table 2:** Level of attendance or provision of samples (%) at study sites for school children and adults relative to receiving incentives, compensation, re-imbursement and treatment

Indicator/Description	School children	Adults	Adults CABs Mobilization	Adults compensation	Adults re-imbursement	Adults Incentives	School children Incentive
Number in all study sites Median age (Range) Females % (n)	1,450 12.4 (8 - 17) 54.8% (795)	1,008 38.8 (18 - 63) 60% (605)	1,008	1,008	1,008	1,008	1,450
Provided samples: Urine/feaces %	80	25	35	70	70	80	80
Provided blood samples %	80	45	45	80	80	80	80
Received treatment	70	45	35	80	80	80	80
Received parasitology results %	35	25	35	80	80	80	80

Table 2, summarizes the need for compensation, incentives or re-imbursement through active participation at community level, through passive discussions with researchers and regulatory agents and at different institutions. Since the assessment/observation was standardized at community levels there is no difference between the six groups regarding features such as 'Number of follow-up events per community', 'Average number of participants per event and community', and 'Number of participation during the baseline and subsequently at each follow-up time point'. However, groups differed significantly regarding active participation at the events. School children in organized settings emerging to show high participation at all times at above 80% and 70% participation for parasitology/blood sampling and to receive treatment, respectively. The level of participation at school events was similar across groups, since participation was mandatory for school children in all schools in the study site (Table 2).

Participation group indicators correlated with each other and the estimates indicate that 'Total number of compliance by at least one indicator group' was positively associated with availability of samples of each group (Table 1). The results showed that availability of personal incentive, compensation or re-imbursement were more likely to enhance participation (OR: 3.38; 95% CI: 1.07-7.70) and to access any form of token given by researchers in community (OR: 2.02; 95% CI: 1.44-3.82). Furthermore, even school children from religious sector that do not take treatment was positively associated with increased participation when there is an incentive or a small token (provision of sweets or school writing notebooks) of appreciation (OR: 2.18; 95% CI: 1.17-3.20); an increase from 70% to 80% to receive treatment and a further increase from 35% to 80% encouraged to know their infection status when an incentives was introduced (Table 2).

About 90% (907) of the study participants and guardians expected a reward of reasonable value, in view of the researchers' value and in comparison to what is offered at other sites regardless of economic status of the community. Discussion among researchers at a basic ethics training workshop indicated that 80% (32) believed the decisions on level of incentive should be determined by the local research ethics committees (Table 3). While, the few international research collaborators were of the opinion that reward or compensation should be in accordance with local guidelines, and in line with funding as agreed and documented in the protocol during the design and reviews. The study revealed that participants and guardians were not happy about decision on level and type of incentive, reimbursement or compensation being reached on behalf of study participants without considering their expectations. On considering expectations of reward revealed that researchers should consult CAB members since they represent the community. In contrast to the adult participants and guardians of children involved in studies, who were of the opinion that compensation and incentives should be at individual level. Both the CAB members and study participants expressed that there should be a clear distinction between study incentive and compensation according to individual and community benefits from studies. Finally, CAB members expressed that the regulatory authorities that normally cite concerns unknown to them, often moderate their suggestions.

**Table 3:** Preference of incentives or compensation given to study participants.

Target respondent	Individual award	Monetary award	Community service or as development
Study participant n = 1,008	Award (100%)	Award (100%)	Limited award (<50%)
CABs/Community Leaders n= 73	Limited award (<50%)	None	Award (100%)
Researchers n = 32	Limited award (<50%)	None	Award (100%)
International Researchers n = 5	Award according to IRB/NRA recommendation	Award if sponsor agrees	Award (100%)
Institutional Review Board/ National Regulatory Authority n = 4	Limited award (<50%)	Limited award (<50%)	Award (100%)

## Discussion

Realization of ethical principles by study participants in communities starts with researchers as they draw out the proposed protocol. These are upheld or authenticated by the ethics committees through reviewers as the study protocol goes through assessment and the approvals process. Further, data safety and monitoring boards (DSMBs) try to observe that ethical guidelines are maintained and there is no prejudice while testing the study hypotheses. While the regulatory authorities (e.g. Medicines Control Authorities, Medical Research Councils, etc.) verify that ethical principles are adhered to and practised during the conduct of the studies. However, governments through the ministry of health and other ministries overseeing research, sometimes take advantage of the research activities to fulfil their own political promises using resources supposed to be research incentives. Very often it is not rare to find some policy makers twisting the regulations to achieve certain goals for the communities. While rarely advocacy groups including NGOs represent the community leaders and the voiceless participants.

Lack of empowerment exposes African research participants and even African researchers and institutions to exploitation, coercion, enticement and inducement that would compromise overall voluntariness, and even upholding fairness is research studies. The sponsor and the investigator must take every effort to ensure that the research is responsive to the health needs and priorities of the population or community in which it is to be carried out. If the capacity is lacking, steps must be taken to strengthen the oversight mechanisms [4]. Usually, some key players are easily identified with major responsibilities in research, however, the concern in determining the respect for the research participant is often over looked. Most guidelines refer to research participants as mere study components to be protected disregarding the need for rewards and individual benefits. The hierarchy in research authority and all responsible overseers should understand the demands and needs of the communities they protect. Research in resource limited areas need to have prescriptive guidelines that accounts for individual desires for rewards. The research participant is a living individual from whom a researcher obtains data and specimens. The investigation is performed on the research participant that means the individual is central to the activities. Researchers and all responsible authorities have ethical and legal obligations to protect and satisfy human participants universally [3,13].

The fundamental ethical principle of justice requires fairness or entitlement that is giving to each what is due. Human beings are morally equal and should be treated as such regardless of colour, creed, race or religion including economic status. This principle of justice demands fairness in treatment of individuals and communities as such there should be equitable distribution of the burden and benefits of research. Important implications for such issues include rewards to participants during the study and post-study benefits. Generally, the communities in resource-constrained areas still do not enjoy the fruits of study participation at an individual level. The fundamental principle of autonomy requires that the wishes and choices of an individual be respected [2]. Individuals in research studies must be their own masters and can act or make free choices and take decisions without constraint of another. This is rare where no individual opportunity is given to make an informed decision to ask if not demand for reward, rather level and the regulatory authorities presume the type of reward, and very often the principle of divide and rule is applied. No discussion is permitted even though consent is obtained. Most resource-constrained communities do not exercise their demand rights but are entangled in mob participation that is taken advantage of by the area regulatory authorities under the pretext of representing the participants.

Most communities involved in studies or clinical trials are presumed to understand the essence of research. Even with these assumptions, participants should be given adequate information and explanation hence this implies that they volunteer to be objects of some experiments. Even though being aware that it is not an obligation to participate in the research, most resource constrained communities and individuals flow together without demanding or exercising the right of being free to refuse participation. The success of research is highly dependent on the willingness and cooperation of the participants during the protocol activities, by providing information or specimens. Informed consent plays an important part in this regard [1,2,7]. The responsibilities often are weighted on the investigator even though there are considerable responsibilities on the research participant. In most instances, community representatives are a major player in decision making in as far as research participation. Researchers and trial sponsors need to consult communities through transparent and meaningful participatory process that involve participants during the early stages in the design, development, implementation and monitoring of the study activities [18]. Consultations maybe arranged through local community leaders such as headmen, chiefs, community health workers and local civic leaders. Most communities where research activities have been conducted, certain mechanisms have been established for community engagement by establishing community advisory boards.

Researchers or investigators have mammoth responsibilities that include extreme caution on the vulnerable populations. The Helsinki Declaration mentions the observation of the benefits to the community [1]. The Investigator must make evaluation on the benefits to the communities and or individual. The sponsor or investigator should make every effort to ensure the work is responsive to the health needs and the priorities of the population or community in which the study is being conducted. After the study or intervention, the knowledge generated should be made available for the benefit of the population or community [1-4,7].

One challenge of assessing the effectiveness of biomedical field research implementation is the lack of a reliable, unbiased and accepted indicator to measure participation. Compliance with the biomedical research programme and intervention (e.g. epidemiology project, clinical trials or testing clinical tools) is an important indicator of a successful implementation strategy. To our knowledge, none of the several studies that measured study participation in relation to compensation and giving out incentives as effectiveness to improve participation assessed determinants of compliance directly. To date, the most common end-points used to assess compliance rely on statistics deducted from successful follow-up as the indirect observation of willingness to participate and these indicators are often assessed once, usually at the end of the intervention, and the reliability of these indicators is unknown. Self-reported compliance in the context of an interview is known to produce inflated results due to reporting bias. In this study we use six measures of direct observation and researcher tallying attendance from sample availability to create a score to classify participation according to 'willingness to participate' by being present at examination and interview day, and 'provision of samples' as required on appropriate days. However, this approach to participation and availability of sample classification uses components that can readily indicate magnitude of willingness. Agreeing to be part of the study forces the investigator to subjectively determine the acceptance of the study by the community. There is a need for objective methods to classify participants into distinct willing groups and also to consider views of the CABs and other community leaders.

In this article we present a detailed analysis of research study compliance among participants from resource limited settings who participated in different community based studies in rural Zimbabwe. The assessment detected a highly statistically significant demand for incentive in school children and in adults with an overall compliance of above 80% based on both community- health worker assessment and the research staff. Here, we use research data collected over a number of studies whose participant compliance was monitored by CABs and the study researchers to objectively deduce participation. We then use the classified groups to describe the participation determinants that are associated with the general researcher-participant attitude in resource-constrained setting.

Systematic reviews of biomedical studies and clinical interventions in developing countries reveal that majority of the global inhabitants have somehow been reached by certain forms of research programme that demand their participation. Further, from empirical research data, the world is now becoming a village in as far as being accessed by study programs is concerned. The global research events indicate that it is becoming increasingly difficulty to isolate communities from what was practiced in other areas during conduct of research programs. Even justification for grossly different levels of incentives, compensation or reimbursement can no longer continue, as the world becomes a global village. There is need for collective consideration of both personal and community rewards in future studies to be conducted in resource-constrained communities. Information available indicates a projected fear that recruitment in future may be a challenge, now that almost every community has somehow been reached and participated in some form of research studies. A major concern is that study participation rewards should be internationally pegged regardless of different economic status of the individuals or communities.

The challenge for incentives, compensation or even reimbursement is addressed unequally between regions and development status of the community or country. Resource constrained communities welcome many forms of studies regardless of the exploitation levels, due to poverty. Most of these communities are not empowered to air out their demands. Further, in such communities there is no recourse to challenge irrational health research policies and administrative decisions. If research intellectual and legal rights can be shared equitably between researchers and their institutions - what is preventing individualized benefits to study participants? We characterized six distinct participation groups in studies conducted over 6 years among participants of school- and community-based studies in rural Zimbabwe. Participation characteristics that were most strongly associated with the categorized groups include giving incentives, the level of compensation or reward for time taken to participation. These three forms of study participant benefit were strongly associated with participation. Promotion of efforts to give the study participants something would more easily encourage participation, and presumably reduce recruitment time.

Our findings suggest that the motivation to provide samples and to participate; even for treatment requires some form of rewards. In addition, higher compliance and obtaining samples was associated with the frequency of issuing out some token of appreciation to promote individual attendance at sample collection time point. It is likely that eager participants providing the biological samples are more interested in participating at the related promotional events and with incentives. Applying the theory and belief of due influence if incentives are used, has no place in designing studies in the modern research programme. These coherent findings on the motivating factors for participation underscore the importance of determining form and level of incentive for the participants prior to implementing the project. In combining objective indicators that measured visible signs of willing participation (e.g. provision of biological samples or being present to give the samples especially blood samples for immunological work) with proxies indicative of responsive to CABs encouragement and the presence of the biological samples collected at the required time point increased the quality of measurement and reduced the potential for reporting bias. The CABs evaluation on compliance generated much lower willingness rates than research staff on actual sample availability observation. This underscores the potential for bias in situations where community based staff as members of the CABs evaluate their own work through compliance after mobilization. Our results highlight the importance of choosing independent staff and a valid and responsive indicator to assess willingness and compliance and to draw conclusions about the need or effectiveness of incentives in intervention programme.

Despite an intensive baseline and continuous mobilization campaign carried out by research teams and CABs members, we observed 35% overall compliance without any reward given out at subsequent follow-up time point. However, when incorporating a simple token or reward in the form of dried fish and cooking oil, participation increased to over 70%. While during the follow-up when the rewards were not available, there was a reasonable response to participation on the first day but when participant realized that nothing was being given out, the attendance dropped drastically. Introduction of a small token or reward during follow-up showed an increase to treatment uptake, even when giving out treatment with sweetened orange crush juice to a sector of school children from a religious group that does not accept treatment. Our findings suggest that biomedical research programme would benefit from reassessing the core requirement for compliance. According to information from resourced communities, there are stark differences in marketing messages and approaches to reach the critical fraction of the population to participate in such studies. Our analysis identified some characteristics associated with increased willingness to participate, after receiving a small token or reward, indicating the potential to draw community members to the study. Most of the concerns can be assessed and addressed during the writing up of the protocols and incorporated during marketing and promotion strategies targeting the participants and informing on the personal benefits and rewards from participation. Based on the characteristics that we measured, it was clear to differentiate the willing participant from 'incentive driven participation (Table 2). In the study, the population of the participant groups included the most marginalized rural communities by observable characteristics: they were poor, lived further from health services centres, rarely had enough daily resources, the communities have high prevalence of neglected tropical diseases. We give evidence of the need to include individual token of appreciation. But the agents involved in designing and reviewing the protocols rarely would agree to reward participants in whatever form that the communities would appreciate.

In the resource constrained areas context, programme planning may benefit from assessing easy measurable factors like the individual desires and community expectations, a large proportion of population subgroups in these poor communities that can be targeted for biomedical research sites do not have excessive demands for compensation or rewards at individual level. Those insights supported by our data are consistent with recommendations for a successful rollout of clinical trials programme deriving from other previous studies. Central government suggested levels of appreciation for the communities are not in line with what the community and individuals expect and sometimes rarely would the communities receive these contributions from the researchers. Normally, it is not uncommon that resources from research programme get diverted or replaces government responsibilities, hence individual senior government officials mat benefit from such confusion. Individual benefits should be considered separate from government responsibilities where these are required. As a result, regulatory authorities very often propose making use of community rewards from study programme for community developments, thereby portraying differences to studies conducted in resourceful areas. The difference in not permitting the rewards to an individual is beyond any reasonable thinking besides among those who wish to deny these communities their dues from participation in studies.

There are limitations to this study. The participating communities were not homogenous regarding preexisting infrastructures, previous exposure to research campaigns and biomedical programme, as well as political support to participate in the study. Finally, data on the willing to participate and comparable time points where a reward was introduced, may somehow differ because (i) the indicator was implemented by different groups, and (ii) availability of samples and presence to uptake treatment in non-invasive biomedical studies. We believe such measurements somehow enhanced the reliability of willingness to participate due to a direct visible benefit and none invasiveness of the study procedure.

## Conclusions

Analyses of implementation effectiveness and the willingness to participate in research programme are rarely published. Our findings suggest that individuals from resource-constrained settings are marginalized in deciding their fate and desires for their participation in research studies. This finding suggests how researchers could identify from the communities where studies are to be conducted, the general wish for rewards of the populations most likely to encourage participation in studies. Introduction of such rewards would be greatly beneficial to clinical, epidemiological and other similar studies by reducing recruitment time frame in reaching desired sample sizes. The key finding here is that communities feel marginalized in decision making. There is no clear conclusion on the view of the stakeholders on participation incentives, compensation and re-imbursement. The subjects need awareness on their rights under the principles of Universal declarations of Bioethics and Human Rights and other international normative instruments on life sciences research ethics.

## Competing interests

The authors declare that they have no competing interests.

## Authors' contributions

TM, NM, DD and PN conceived the idea and developed the design for the study. TM wrote the original draft manuscript, and incorporated revisions from each of the co-authors. NM and DD contributed to the conception and design of the manuscript and conducted the statistical analysis. TM and NM coordinated and supervised data acquisition. All authors read and approved the final manuscript.

## Declarations

Publication of this supplement has been funded by the College of Health Sciences and the Research Office at the University of Kwazulu-Natal.

This article has been published as part of *BMC Medical Ethics *Volume 14 Supplement 1, 2013: Selected papers from the 3rd Ethics, Human Rights and Medical Law Conference (3rd EHMRL). The full contents of the supplement are available online at http://www.biomedcentral.com/bmcmedethics/supplements/14/S1.

## References

[B1] World Medical Association Ethical principles for medical research involving human subjects 2008 http://www.wma.net Seoul Korea: Declaration of Helsinki 59th WMA General assembly

[B2] Belmont Report Ethical principles and guidelines for the protection of human subjects of research http://www.hhs.gov/ohrp/humansubjects/guidance/belmont.html Report of the National commission for protection of the human subjects of biomedical and behavioral research; 1979.

[B3] Nuffield Council on Bioethics The ethics of research related to healthcare in developing countries 2002 Nuffield council

[B4] International ethical guidelines for biomedical research involving human subjects 2002 http://www.cioms.ch/publications Geneva: Council for international organization of medical sciences (CIOMS) in collaboration with World Medical Organization (WHO) 14983848

[B5] Mduluza T Mduluza T, Chima SC and Nsimba S Towards community engagement A Gateway to Biomedical Research in Africa 2007 New York: Nova Science Publishers 147 172

[B6] Ndebele P Mfutso-Bengo J Mduluza T Compensating clinical trial participants from limited resource settings in internationally sponsored clinical trials: a proposal Malawi Med J 2008 14 2 42 5 19537431 10.4314/mmj.v20i2.10955 PMC3345670

[B7] Operational guidelines for ethics committees that review biomedical research 2000 Geneva: World Health Organization (WHO)

[B8] Hughes JR Exclusion of "Noncompliant" individuals from clinical trials Controlled Clinical Trials 1993 14 176 7 10.1016/0197-2456(93)90019-A 8500306

[B9] The Belmont Report | HHS.gov 2011 http://www.hhs.gov/ohrp/humansubjects/guidance/belmont.html

[B10] Council of Europe - ETS no. 164 - Convention for the Protection of Human study participants" 2003

[B11] Nuffield Council on Bioethics. The ethics of research related to healthcare in developing Countries 2002 Latimer Trend Group. UK

[B12] Council of International Organizations of Medical Sciences International Ethical Guidelines for Biomedical Research involving human subjects 1993

[B13] WHO technical report Guidelines for good clinical practice (GCP) for trials on pharmaceutical products World Health Organization technical report series No. 850, 1995, annex 3

[B14] Nyika A Kilama W Chilengi R Tangwa G Tindana P Capacity building of ethics reviews committees across Africa based on the results of a comprehensive survey Developing World Bioethics 2008 14 8731 10.1111/j.1471-8847.2008.00243.x 20021494

[B15] Mudur G John Hopkins admits scientist used Indian patients as guinea pigs BMJ 2001 14 7323 PMC1121689 11719406

[B16] Dyer O GP suspended for enrolling patients in drug trials without consent BMJ 2003 14 304 10.1136/bmj.326.7384.304 PMC1125182 12574041

[B17] Beauchamp LT Childress FJ Principles of Biomedical Ethics 2001 5 Oxford: Oxford University Press

[B18] Molyneux CS Peshu N Marsh K Trust and Informed consent: Insights from community members on Kenyan Coast Soc Sci Med 2002 14 1463 1473 10.1016/j.socscimed.2004.11.073 16005781

[B19] Nyika A Kilama W Chilengi R Tangwa G Tindana P Ndebele P Ikingura J Composition, raining needs and independence of ethics review committees across Africa: are the gatekeepers rising to the emerging challenge? J Med Ethics 2009 14 189 193 10.1136/jme.2008.025189 19251972 PMC2643018

[B20] Weijer C Miller PB Protecting communities in Pharmacogenetics and Pharmacogenomics research Pharmacogenomics J 2004 14 9 16 14647406 10.1038/sj.tpj.6500219

[B21] World Health Organization. The Global Burden of Disease World Health Organization 2004 Geneva

[B22] Doyal L Gender and 10/90 Gap in Health Research. Bull World Health Organization 2008 14 PMC2585920 15112002

[B23] Singh JA Using the courts to challenge irrational health research [olicies and administration decisions Acta Tropica 2009 14 553 562 10.1016/j.actatropica.2009.07.035 19665981

[B24] Pinheiro CP Drug donations: what lies beneath Bull World Health Organization 2008 14 8 580 584 10.2471/BLT.07.048546 PMC2649461 18797609

[B25] Lavery VJ Grady C Wahl RE Emmanuel JE Ethical issues in International Biomedical Research. A case Book 2007 Oxford: Oxford University Press

[B26] Frost LJ Reich MR Access: How Do Health technologies get to the poor people in the poor countries? Harvard Centre for population and Development Studies 2008 Cambridge, Massachussettes

[B27] Appleby LA Nausch N Midzi N Mduluza T Allen JE Mutapi F Sources of heterogeneity in human monocyte subsets Immunol Letters 2013 14 32 41 10.1016/j.imlet.2013.03.004 PMC3684771 23557598

[B28] Mduluza T Gori E Chitongo P Midzi N Ruwona T Soko W Mlambo G Mutambu SL Kumar N Analysis of TNF-α and IL-10 gene polymorphisms in Zimbabwean children exposed to malaria African J of Biotech 2013 1034 1039

[B29] Nausch N Louis D Lantz O Peguillet I Trottein F Chen IY Appleby LJ Bourke CD Midzi N Mduluza T Mutapi F Age-Related Patterns in Human Myeloid Dendritic Cell Populations in People Exposed to Schistosoma haematobium Infection PLoS Negl Trop Dis 2012 14 9 e1824 10.1371/journal.pntd.0001824 23029585 PMC3459871

[B30] Reilly L Nausch N Midzi N Mduluza T Mutapi F Association between Micronutrients (Vitamin A, D, Iron) and Schistosome-Specific Cytokine Responses in Zimbabweans Exposed to Schistosoma haematobium J Parasitol Res 2012 128628 22523639 10.1155/2012/128628 PMC3317203

[B31] Rujeni N Nausch N Bourke CD Midzi N Mduluza T Taylor DW Mutapi F Atopy Is Inversely Related to Schistosome Infection Intensity: A Comparative Study in Zimbabwean Villages with Distinct Levels of *Schistosoma haematobium *Infection Int Arch Allergy Immunol 2012 14 288 298 10.1159/000332949 22398631 PMC3398828

[B32] Midzi N Mtapuri-Zinyowera S Sangweme D Paul NH Makware G Mapingure MP Brouwer KC Mudzori J Hlerema G Chadukura V Mutapi F Kumar N Mduluza T Efficacy of integrated school based de-worming and prompt malaria treatment on helminths -Plasmodium falciparum co-infections: A 33 months follow up study BMC Int Health Hum Rights 2011 14 9 18 10.1186/1472-698X-11-9 PMC3141662 21696629

[B33] Midzi N Mtapuri-Zinyowera S Mapingure MP Paul NH Sangweme D Hlerema G Mutsaka MJ Tongogara F Makware G Chadukura V Brouwer KC Mutapi F Kumar N Mduluza T Knowledge attitudes and practices of grade three primary schoolchildren in relation to schistosomiasis, soil transmitted helminthiasis and malaria in Zimbabwe BMC Infect Dis 2011 14 11 169 10.1186/1471-2334-11-169 PMC3141409 21668948

[B34] Mutapi F Rujeni N Bourke C Mitchell K Appleby L Nausch N Midzi N Mduluza T Schistosoma haematobium treatment in 1-5 year old children: safety and efficacy of the antihelminthic drug praziquantel PLoS Negl Trop Dis 2011 14 5 e1143 10.1371/journal.pntd.0001143 21610855 PMC3096601

[B35] Mduluza T Mutapi F Ruwona T Kaluka D Midzi N Ndhlovu PD Similar cellular responses after treatment with either praziquantel or oxamniquine in Schistosoma mansoni infection Malawi Med J 2009 14 4 176 82 21174933 10.4314/mmj.v21i4.49642 PMC3345748

[B36] Sangweme DT Midzi N Zinyowera-Mutapuri S Mduluza T Diener-West M Kumar N Impact of schistosome infection on Plasmodium falciparum Malariometric indices and immune correlates in school age children in Burma Valley, Zimbabwe PLoS Negl Trop Dis 2010 14 11 e882 9 10.1371/journal.pntd.0000882 21085468 PMC2976682

[B37] Midzi N Mtapuri-Zinyowera S Mapingure MP Sangweme D Chirehwa MT Brouwer KC Mudzori J Hlerema G Mutapi F Kumar N Mduluza T Consequences of polyparasitism on anaemia among primary school children in Zimbabwe Acta Trop 2010 14 1-2 103 11 10.1016/j.actatropica.2010.02.010 20175980

[B38] Kjetland EF Hove RJ Gomo E Midzi N Gwanzura L Mason P Friis H Verweij JJ Gundersen SG Ndhlovu PD Mduluza T Van Lieshout L Schistosomiasis PCR in vaginal lavage as an indicator of genital Schistosoma haematobium infection in rural Zimbabwean women Am J Trop Med Hyg 2009 14 6 1050 5 10.4269/ajtmh.2009.09-0081 19996436

[B39] Mtapuri-Zinyowera S Midzi N Muchaneta-Kubara EC Simbini T Mduluza T Impact of solar radiation in disinfecting drinking water contaminated with Giardia duodenalis and Entamoeba histolytica ⁄dispar at a point-of-use water treatment J Appl Microbiol 2009 106 847 852 19191972 10.1111/j.1365-2672.2008.04054.x

[B40] Midzi N Butterworth AE Mduluza T Munyati S Deelder AM van Dam GJ Use of circulating cathodic antigen strips for the diagnosis of urinary schistosomiasis Trans R Soc Trop Med Hyg 2009 14 1 45 51 10.1016/j.trstmh.2008.08.018 18951599

[B41] Mlambo G Mutambu SL Mduluza T Soko W Mbedzi J Chivenga J Lanar DE Singh S Carucci D Gemperli A Kumar N Antibody responses to Plasmodium falciparum vaccine candidate antigens in three areas distinct with respect to altitude Acta Trop 2006 14 1-2 70 8 10.1016/j.actatropica.2006.09.012 17113021

[B42] Ndhlovu PD Mduluza T Kjetland EF Midzi N Nyanga L Gundersen SG Friis H Gomo E Prevalence of urinary schistosomiasis and HIV in females living in a rural community of Zimbabwe: does age matter? Trans R Soc Trop Med Hyg 2007 14 5 433 8 10.1016/j.trstmh.2006.08.008 17064746

[B43] Kjetland EF Ndhlovu PD Gomo E Mduluza T Midzi N Gwanzura L Mason PR Sandvik L Friis H Gundersen SG Association between genital schistosomiasis and HIV in rural Zimbabwean women AIDS 2006 14 4 593 600 10.1097/01.aids.0000210614.45212.0a 16470124

